# The effectiveness of self-care and lifestyle interventions in primary dysmenorrhea: a systematic review and meta-analysis

**DOI:** 10.1186/s12906-019-2433-8

**Published:** 2019-01-17

**Authors:** Mike Armour, Caroline A. Smith, Kylie A. Steel, Freya Macmillan

**Affiliations:** 1 NICM Health Research Institute, Western Sydney University, Sydney, Australia; 2 School of Science and Health, The MARCS Institute, Western Sydney University, Sydney, Australia; 3 School of Science and Health, Western Sydney University, Sydney, Australia

**Keywords:** Self-care, Period pain, Exercise, Heat, Acupressure

## Abstract

**Background:**

Menstrual pain is very common amongst young women. Despite the significant impact that menstrual pain has on academic attendance and performance, social activities and quality of life, most young women do not seek medical treatment but prefer to use self-care; commonly OTC analgesic medications and rest. Many women do not get significant pain relief from these methods, therefore other low cost, easy to learn self-care methods may be a valuable approach to management.

This review and meta-analysis examines the evidence for participant lead self-care techniques.

**Methods:**

A search of Medline, PsychINFO, Google Scholar and CINAHL was carried out in September 2017.

**Results:**

Twenty-three trials including 2302 women were eligible and included in the meta-analysis. Studies examined self-delivered acupressure, exercise and heat as interventions. Risk of bias was unclear for many domains. All interventions showed a reduction in menstrual pain symptoms; exercise (g = 2.16, 95% CI 0.97 to 3.35) showed the largest effect size, with heat (g = 0.73, 95% CI 0.06 to 1.40) and acupressure (g = 0.56, 95% CI 0.10 to 1.03) showing more moderate effect sizes. Exercise (g = 0.48, 95% CI 0.12 to 0.83) and heat (g = 0.48, 95% CI 0.10 to 0.87), were more effective than analgesics in reducing pain intensity, whereas acupressure was significantly less effective (g = − 0.76, 95% CI -1.37 to − 0.15).

**Conclusion:**

Exercise showed large effects, while acupressure and heat showed moderate effects in reducing menstrual pain compared to no treatment. Both exercise and heat are potential alternatives to analgesic medication. However, difficulties in controlling for non-specific effects, along with potential for bias, may influence study findings.

## Background

Menstrual disorders are highly prevalent amongst women, and most commonly feature period pain and mood disturbances. Primary dysmenorrhea (period pain) affects around three quarters of all women during their reproductive life, and is especially common in young women in their teens and early adult life [1], with around 90% of Australian adolescents experiencing menstrual pain [2, 3]. Primary dysmenorrhea is defined as menstrual pain in the absence of any organic cause with the pain commonly starting within three years of menarche (the first menstrual period) [4]. Primary dysmenorrhea’s characteristic symptom is crampy, colicky spasms of pain below the belly button, occurring within 8–72 h of menstruation, and peaking within the first few days as menstrual flow increases [5]. In addition to painful cramps, many women with dysmenorrhea experience other menstrual related symptoms including back and thigh pain, headaches, diarrhoea, nausea and vomiting [5].

The largest contributing physiological factor in primary dysmenorrhea is increased amounts of prostaglandins present in the menstrual fluid [6]. Prostaglandins, especially PGF2_a_, stimulate myometrial contractions reducing uterine blood flow and causing uterine hypoxia. This hypoxia is responsible for the painful cramping that characterises primary dysmenorrhea [6, 7].

Primary dysmenorrhea is responsible for a decrease in quality of life [8–10], absenteeism from work or school [7], reduced participation in sport and social activities [11], altered pain perception and sleeping problems [12].

Consensus guidelines [13] and reviews of the evidence [5, 6, 14–16] suggest that non-steroidal anti-inflammatory medications (NSAIDs) are an effective first line treatment for primary dysmenorrhea. The combined oral contraceptive (COC) pill is a common second line of treatment for primary dysmenorrhea [5, 7], though it may be used as a first line treatment when long-term contraception is required [4]. While NSAIDs and COC are effective for many women, approximately 25% of women have pain that is refractory to either of these standard treatments [17, 18]. In addition, cultural differences also affect the usage of analgesics and the oral contraceptive pill, with Chinese women using significantly less NSAIDs or the oral contraceptive to control their menstrual pain than Australian women [19].

Most women manage their symptoms with primarily over the counter (OTC) pain medications (e.g. ibuprofen and paracetamol/acetaminophen), and self-care including rest and the application of heat, rather than seeking medical advice [8, 9, 11, 20–27]. Lack of satisfactory pain relief and effective medical interventions in primary dysmenorrhea leads to an uptake of self-care strategies by women [28]. Complementary, non-pharmacological or traditional medicine usage (such as herbal medicines or dietary changes) are often a significant component of self-care [28, 29]. Many women already use various forms of non-pharmacological techniques to manage their menstrual pain [24, 30, 31]. This is in addition to, or instead of, pharmaceutical pain relief due to either a lack of perceived effectiveness of these medications [23, 27, 28] or a dislike of using analgesic medication due to concern over side effects [32].

However one key barrier in managing menstrual pain, whether it be pharmacological or non-pharmacological treatment, is that the intervention needs to be affordable, both in terms of time (in terms of attending appointments and scheduling) and cost [33]. Non-pharmacological self-care techniques or lifestyle interventions, either physical or psychological, that can be practiced by women themselves such as exercise (including yoga and Pilates), heat, meditation, aromatherapy, self-massage or acupressure may fulfil these criteria, allowing women to potentially reduce their menstrual pain and need for analgesics, and improve their health-related quality of life. A recent Cochrane review examined dietary and herbal supplements (such as fish oil) for dysmenorrhea [34] but there are no recent reviews examining participant lead self-care interventions for primary dysmenorrhea.

### Objectives

The aim of this review was to determine the effectiveness of participant lead self-care techniques and lifestyle interventions on menstrual pain intensity, duration, and analgesic usage in women with primary dysmenorrhea.

## Methods

Preferred Reporting Items for Systematic Reviews and Meta-Analyses (PRISMA) guidelines were adhered to throughout this review [35].

### Search strategy

Databases searched included Medline, PsychINFO, Google Scholar and CINAHL from 1 August 1997 to 1 September 2017 using the keywords “self-care” OR “lifestyle” OR “breathing” OR “meditation” OR “exercise” OR “yoga” OR “acupressure” OR “massage” OR “aromatherapy” OR “mindfulness” AND “dysmenorrhea” OR “period pain” using the Boolean ‘AND/OR’ operators. These keywords are a modification of those used in our recent Cochrane review on pain management in labour [36]. Both MESH and Non-MESH terms were included in this search. Papers that either had English full text or where an English translation was available from our recent Cochrane systematic review [37] were included. Reference lists of full text papers were searched, and any relevant articles identified were screened.

### Eligibility criteria

For the purposes of this review, eligible interventions comprised of participant lead self-care and lifestyle interventions defined as physical, including exercise, or psychological techniques that women could administer themselves and were considered to be low-risk. The American College of Sports Medicine (ACSM) definition for exercise was used, where “exercise is physical activity characterized by using planned and structured repetitive movements to increase or maintain physical fitness” [38]. Techniques such as yoga, meditation, mindfulness or acupressure, which could be learned (either in person or online) and independently self-administered were included, as was self-massage, but not massage that was delivered solely by a therapist or researcher. Acupressure was eligible when it was delivered by the participant for at least some of the trial period (e.g. was delivered and taught by a therapist for the first month), but not if only delivered by a therapist, researcher or other external party.

Primary dysmenorrhea is a diagnosis of exclusion, and there is no conclusive test to diagnose primary dysmenorrhea. We did not include any trials that specifically included women with diagnosed secondary dysmenorrhea (such as PCOS or endometriosis). Randomised and quasi-randomised trials were included but cross-over trials, due to the cyclical nature of primary dysmenorrhea and the unknown wash-out period of most non-pharmacological interventions, were not included. For inclusion outcome measures needed to include either a direct measure of pain intensity or severity (such as a visual analogue scale (VAS) or numeric rating scale (NRS)), or a composite score using a scale such as the Moos Menstrual Distress Questionnaire (MMDQ) or the Short form McGill Pain Questionnaire (SF-MPQ). At least one of the comparator groups had to be a sham/placebo treatment, analgesic medication, oral contraceptive pill or usual care/no treatment.

### Data extraction

Two authors extracted the data independently and a third author (MA) resolved any disagreement. Where data was missing or unclear, the study authors were contacted via email by the authors to request the missing data be provided. Authors were contacted twice over a 6-week period. If no response was had in that time, the data was marked as missing.

Data was extracted on all of the following outcomes (if reported):

- Menstrual pain intensity or severity (using VAS or NRS)
- Composite pain or symptom score (such as MMDQ)
- Menstrual pain duration
- Analgesic usage
- Absenteeism
- Adverse events

When multiple data points were available post intervention (e.g. end of intervention or one month follow up), end of intervention data was used as the primary time point. If follow-up data was available it was categorised into short term (1 to 3 months), medium term (3–6 months) and long term (6 months or more). Where there were multiple active intervention groups data was combined from self-care treatment arms into one group as per Cochrane guidelines [39]. When multiple scales were used for pain intensity, preference was given to VAS or NRS over composite scores such as MMDQ that included non-pain related components, and therefore the composite score was not included in the meta-analysis if VAS or NRS scales were available. A narrative description of study characteristics (including intervention and control group descriptions, study location) was synthesised.

### Risk of bias

Included studies were also assessed using the Cochrane Collaboration’s Risk of Bias tool [39]. This examined study quality in six areas of trial design (sequence generation, allocation sequence concealment, blinding of participants and personnel, blinding of outcome assessment, incomplete outcome data, selective outcome reporting), ranking each area as high, low or unknown for risk of bias. Risk of bias assessment was undertaken by two reviewers independently (MA, CS, KS, FM). Any disagreements were resolved by discussion.

### Meta-analysis

Random-effects meta-analyses were conducted using Comprehensive Meta-Analysis software (Version 2). Intervention effect sizes were pre-post changes between intervention and control groups for the primary outcome measure (menstrual pain intensity/severity) and were calculated using Hedges’ g statistic [40], along with 95% confidence intervals (CIs) around the estimated effect-size. If pre-intervention scores were not available post treatment changes only between intervention and control groups were used. Pooled effect sizes were calculated using either menstrual pain intensity or composite pain scores (such as MMDQ) for each intervention type with > 2 studies, using random effects models. No study reported a pre and post-test correlation, therefore we assumed a conservative correlation of 0.7 for the primary outcome. Effect sizes were categorized as small (0.2–0.4), medium (0.4–0.8), or large (greater than 0.8) as per Cohen (1988) [41]. Statistical heterogeneity between studies was quantified using Cochran’s Q and *I*^*2*^ statistic, both of which provide estimates of the degree of heterogeneity resulting from between-study variance, rather than by chance. Cochrane’s Q with *p*-value of < 0.05 was classified as significant heterogeneity, and *I*^*2*^ of more than 75% was considered to indicate high level heterogeneity, *I*^*2*^ of 50–75% as indicative of substantial heterogeneity, and an *I*^*2*^ of less than 40% as low heterogeneity.

Publication bias was tested using the Begg and Mazumdar test, with a p-value < 0.05 suggesting the presence of bias [42, 43]. Where significant bias was detected, a Duval and Tweedie trim-and-fill analysis [44] was conducted to re-calculate the pooled effect size after removing any studies which may introduce publication bias (i.e., small studies with large effect sizes from the positive side of the funnel plot). Additionally, a “fail-safe N” was used to account for the file draw problem [45] estimating the number of non-significant unpublished trials which would be needed to cause the observed *p* value to exceed 0.05.

Pre-planned subgroup analyses were conducted to examine whether effects of these interventions differed when comparing them to different control conditions; sham/placebo, analgesics, oral contraceptive pill and usual care/no treatment.

## Results

**Fig. 1:**
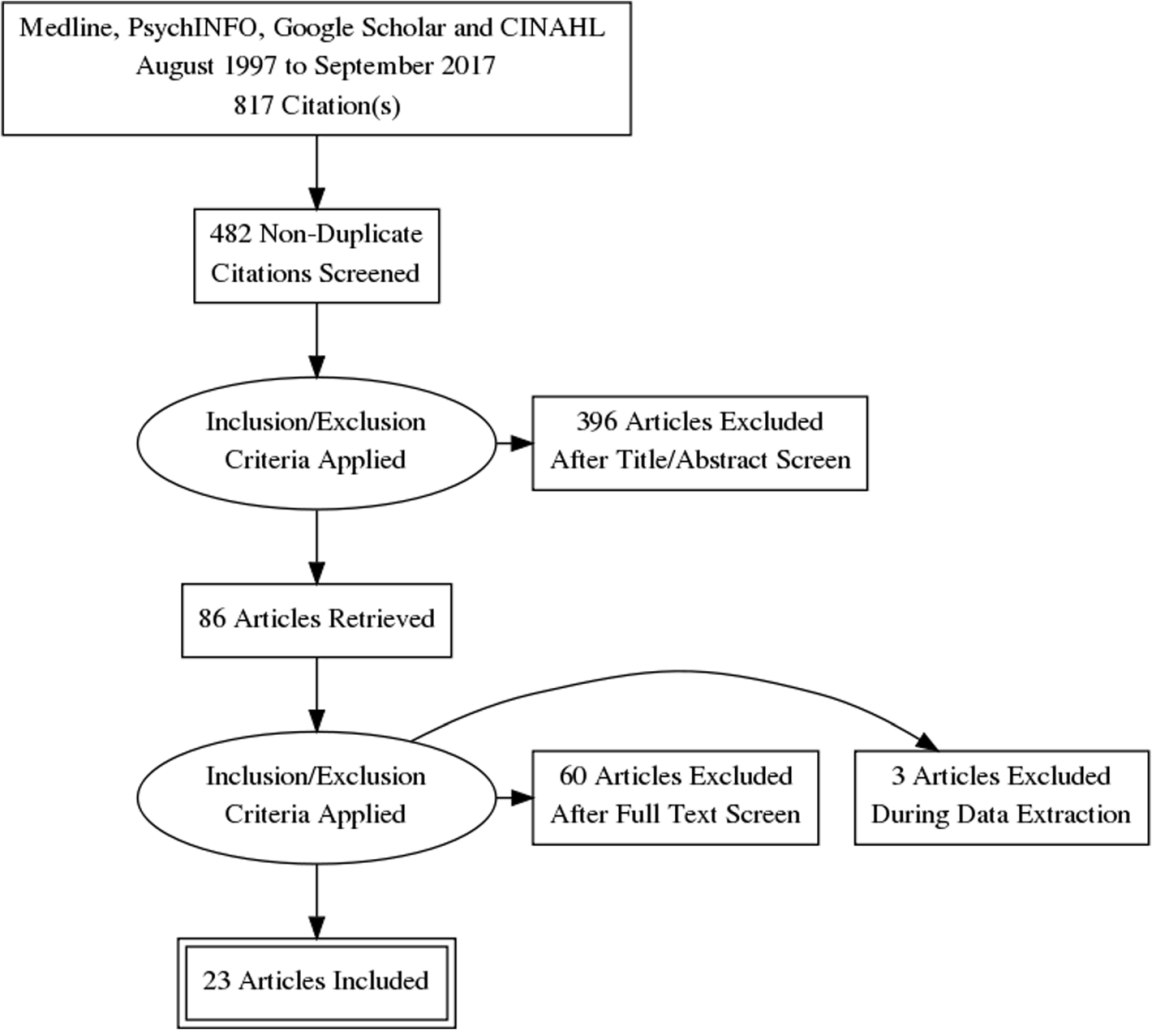
PRISMA flow diagram

Twenty-three trials including 2302 women were eligible and included in the meta-analysis. Figure 1 outlines the search and selection process. Table 1 outlines the characteristics of these studies. No eligible studies on mindfulness or aromatherapy were found.

**Table 1:** Characteristics of included studies

Study ID	Country / number of participants	Intervention	Comparator	Duration	Outcome measures	Summary
Heat						
Akin 2004	USA / 344 women	Active heat wrap (40 degrees C) for eight hours	Oral acetaminophen (paracetamol) 500 mg 4 times per day for one day	1 day, starting on the first day of moderate or greater menstrual pain	Pain relief (categorical 0–5 score, 0 = no pain relief, 5 = complete pain relief) during the eight hours Adverse events	Heat was superior to oral acetaminophen in reducing menstrual pain. One mild AE in the heat group (pink skin)
Akin 2001	USA / 81 women	Heat patch (180cm^2^, 38.9 °C) for 12 h each day, plus placebo pills.	3 comparator groups: 1- Heat patch (180 cm2, 38.9 °C) plus ibuprofen 400 mg, 3 times per day 2- Unheated patch (180 cm2) plus ibuprofen 400 mg, 3 times per day 3- Unheated patch plus placebo pills	2 days – from onset of menstrual pain	Pain relief (categorical 0–5 score, 0 = no pain relief, 5 = complete pain relief) during the 12 h Adverse events	Heated patch plus ibuprofen, heated patch plus placebo pills and unheated patch plus ibuprofen all showed similar pain reductions. The time to onset of pain reduction was shorter in the heated patch plus ibuprofen group compared to the unheated patch plus ibuprofen. No adverse events reported
Potur 2014	Turkey / 193 women	Heat patch (180cm^2^, 38.9 °C, 8 h)	2 comparator groups: 1. Self-medication group (SMG) – single dose of analgesics of participants choice 2. Control group – no medication but could use single dose of oral analgesic if pain was unbearable.	1 day, first day of menses	Pain intensity (VAS) at 4 h and 8 h after starting treatment	*Quasi-randomised based on presentation order.* The group receiving the heat patch reported the greatest pain reduction at 4 h and at 8 h after the start of the intervention.
Rigi 2012	Iran / 150 women	Heat patch (84 cm^2^, 40 °C, 8 h)	Ibuprofen (400 mg) every 8 h as needed	1 Day. First day of menses, once menstrual bleeding started	SF-MPQ at 24 h after starting treatment.	Total pain score as measured by SF-MPQ was not significantly different between groups at 24 h after onset of menses. There was no pre-post comparison. Fchar
Acupressure						
Aghamiri 2005	Iran / 100 women	Acupressure at unspecified points on the abdomen, near the waist and in the leg. 15 min pressure, 15 min rest, then 15 min pressure	Sham acupressure: Same as active group but using ‘off channel’ points.	Frequency and timing of treatment not reported	Pain intensity (VAS) at 180 min following treatment.	Article translated from Farsi. Acupressure group had significantly lower pain scores at 180 min post treatment than sham.
Behbahani 2016	Iran / 120 women	Acupressure at CV4 and CV2. Pressure was applied for 15 s and then 15 s of rest for a total of 20 min.	Two comparator groups: 1. Self-care behaviours. Four 60–90 min sessions, once per week. Isometric exercises were also taught. 2. Ibuprofen group: 400 mg, three times per day.	Acupressure applied over the first two days of menses for two cycles. Comparator groups: Unclear about timing in relation to menses.	McGill pain questionnaire (MPQ) at the end of the 2 month intervention.	Both training and acupressure groups had greater reductions in pain than the ibuprofen group, however no between group comparisons were performed.
Bazarganipour 2010	Iran / 194 women	Acupressure at LR3. Two minutes of firm pressure then two minutes of ‘massage’ on the point. Twenty minutes in total per session. Unclear if alternating sides were used.	Sham Acupressure. Point located at the same level as LR3 but between the 3rd and 4th toes.	Starting three to seven days before menses, for two menstrual cycles.	Andersch and Milsom Scale (0 = no inhibition by pain 3 = work clearly inhibited by pain) during the first menstrual cycle post intervention.	Pain scores decreased in both groups from baseline but the reduction was significantly greater in the acupressure group
Charandabi 2011	Iran / 72 women	Acupressure at SP6. Four seconds of pressure and two seconds without pressure, for five minutes. After five minutes the alternate leg was used, for a total of 20 min per day (4 × 5 min cycles).	Ibuprofen (400 mg) every 6 h as needed	Starting from onset of menstrual pain for two menstrual cycles.	Menstrual symptom severity (5 point Likert scale) for eight symptoms (cramp, headache, back pain, leg pain, depression, irritability, general pain and abdominal pain) Ibuprofen consumption	Menstrual symptom severity had a greater reduction in the acupressure group compared to the ibuprofen group.
Chen 2004	Taiwan / 69 women	Acupressure at SP6. Alternate legs used, two five minute cycles of pressure on each leg (20 min total).	Rest: Participants rested in the school health centre for 20 min.	Once during menses when participants were experiencing cramping	Pain intensity (VAS) at the end of the intervention	No significant difference in pain intensity scores between groups.
Chen 2010	Taiwan / 134 women	Acupressure at LI4 + SP6. Six seconds of pressure, then two seconds without pressure for five minutes per cycle. Two cycles per acupressure point for four cycles (20 min) in total.	Three comparator groups: 1. Acupressure at LI4: Same as active intervention but on LI4 point only. 2. Acupressure at ST36: Same as active intervention but on LI4 point only. 3. Rest: Participants rested in the school health centre for 20 min.	Every day, for the first three days of menses for six menstrual cycles.	Pain intensity (VAS) at the end of the six month follow up period. Menstrual symptoms and distress as measured by the Moos MDQ at the end of the six month follow up period	Active group (LI4 + SP6) vs rest was included in the meta-analysis. LI4 + SP6 group had the greatest reduction in pain intensity and menstrual distress compared to all three comparator groups.
Chen 2015	Taiwan / 129 women	Acupressure at SP6, BL32 and LR3. Four seconds of pressure, then two seconds without pressure, repeated 10 times per minute. After five minutes the alternate side of the body was used for another five minutes. Two five-minute cycles per point, for 30 min in total.	Education control: Education on the use of supplements and dietary therapy for dysmenorrhea.	Three times per week for twelve months.	Pain intensity (VAS) at the end of the 12 month follow up period. Menstrual symptoms and distress as measured by the Moos MDQ at the end of the 12 month follow up period	The acupressure group had greater reductions in pain intensity and menstrual distress compared to the education only group.
Kashefi 2010	Iran / 86 women	Acupressure at SP6 for 30 min, once per cycle.	Sham acupressure: Same as active group but using an ‘off channel’ points.	Frequency and timing of treatment not reported	Pain intensity (VAS) at 180 min following treatment.	Article translated from Farsi. Acupressure group had significantly lower pain scores at 180 min post treatment than sham.
Mirbagher-Ajorpaz 2011	Iran / 30 women	Acupressure at SP6. Eight seconds of pressure and two seconds for rest for twenty minutes in total.	Sham acupressure at SP6 (“light touch”), where no pressure was applied to SP6.	Once during menses, timing unclear.	Pain intensity (VAS), at end of intervention, 1,2 and 3 h post intervention.	Acupressure caused a much greater reduction in pain scores from baseline than sham acupressure.
Pouresmail 2002	Iran / 216 women	Acupressure at SP6, LR3, LI4, SP16, ST36. Pressure for two minutes for each point. Total number of minutes unclear.	Two comparator groups: 1. Sham acupressure at two locations on the arms and two on the legs (exact locations not given). Pressure for two minutes for each point. 2. Ibuprofen (400 mg) three times per day for three days.	Starting 24 h before onset of menses for one cycle.	Andersch and Milsom Scale (0 = no inhibition by pain 3 = work clearly inhibited by pain) at end of intervention Pain intensity (VAS), at end of intervention	Pain measured on both VAS and Andersch and Milsom Scale decreased in all groups after intervention, however ibuprofen and acupressure had the greatest reduction.
Wang 2009	Taiwan / 74 women	Auricular acupressure at Liver (CO12), Kidney (CO10) and Endocrine (CO18) points using acupressure seeds under adhesive patch. Points were stimulated 15 times, three times per day.	Sham adhesive patch: Patches placed on same points as active group but no acupressure seed. Points were stimulated 15 times, three times per day in same manner as active group.	20 days.	Moos MDQ at end of intervention (20 days)	Auricular acupressure group had lower MDQ scores at the end of intervention compared to sham.
Wong 2010	Hong Kong / 40 women	Acupressure at SP6. 15 s of pressure followed by 15 s of rest. Repeated 10 times, totalling five minutes per cycle. Two cycles per leg for a total of 20 min per treatment. Performed twice per day upon waking and at bedtime	No treatment – participants were told to rest for 20 min upon waking and at bedtime.	First three days of menses, for three menstrual cycles.	Pain intensity (VAS) at end of intervention McGill pain questionnaire (SF-MPQ) at end of intervention Moos MDQ (MMDQ) at end of intervention	The acupressure group had a significantly greater reduction from baseline in pain intensity, SF-MPQ and MMDQ scores at the end of the three month intervention.
Yeh 2013	Taiwan / 113 women	Auricular acupressure at Shenmen, Liver, Kidney, Internal Genital, Central Rim and Endocrine acupressure seeds under adhesive patch. Points were stimulated for 1 min each point, four times per day	Sham acupressure: Six auricular acupressure points with no expected effects on dysmenorrhea (wind stream, tonsils, trachea, esophagus, internal nose, pharynx and larynx) using acupressure seeds under adhesive patch. Points were stimulated for 1 min each point, four times per day	2 days from onset of menstrual pain	Pain intensity (VAS) at the end of the intervention Moos MDQ at end of intervention	Auricular acupressure group had greater reductions in both pain and MDQ scores from baseline than sham.
Zafari 2011	Iran / 296 women	Acupressure at SP6. Four seconds of pressure and two seconds without pressure, for five minutes. After five minutes the alternate leg was used, for a total of 20 min per day (4 × 5 min cycles).	Ibuprofen (400 mg) at onset of pain and taken every eight hours if needed .	From onset of menstrual pain, number of days per cycle unclear. Two menstrual cycles.	Pain intensity (self-reported), scale unclear.	Both acupressure and ibuprofen groups had reductions in pain from baseline and there was no between group differences.
Exercise						
Abbaspour 2006	Iran / 150 women	Stretching exercises: 1- Lie face up with legs and knees bent perform abdominal breathing about 10 times. 2- Stand holding backs of chair; lift one heel off the floor, then the other, repeat 20 times. 3- Stand holding back of chair then does 5 deep knee bends. 4- While lying on back lift and bring knees to touch chin, 10 times. Performed twice a day for 20 min. Exercise was not performed during menstruation	No treatment	Two menstrual cycles	Pain intensity (VAS), at end of intervention Pain duration (hours) at end of intervention	Both severity and duration of pain decreased significantly from baseline in the stretching group. No change in the control group.
Motahari-Tabari 2017	Iran / 122 women	Stretching exercises: included a five-minute warm up in a standing position and then six belly and pelvic stretching exercise for 10 min. This program was performed for 15 min, three times a week. Exercise was not performed during menstruation	Mefenamic acid (250 mg) every eight hours when in pain.	Two menstrual cycles (8 weeks)	Pain intensity (VAS), at end of intervention Pain duration (days) at end of intervention	Pain intensity and duration significantly decreased in both groups from baseline, with no differences between groups.
Rakhshaee 2011	Iran / 92 women	Yoga postures: Cobra, Cat and Fish postures, one 20 min session per day,	No treatment	Luteal phase of menstrual cycle (14 days) for two menstrual cycles	Andersch and Milsom Scale (0 = no inhibition by pain 3 = work clearly inhibited by pain) at end of intervention Pain duration (hours) at end of intervention	*Quasi-randomised based on location* Pain severity and duration decreased significantly from baseline in the Yoga group, and this was significantly different to the no treatment group.
Shahr-Jerdy 2012	Iran / 179 women	Stretching exercises: Six exercises in the abdominal, pelvic and groin region. Three days per week and two times per day for 10 min. Exercise was not performed during menstruation	No treatment	Two menstrual cycles (8 weeks)	Pain intensity (VAS), at end of intervention Pain duration (hours) at end of intervention Analgesic usage (number of tablets) at end of intervention	*Quasi-randomised based on location.* Stretching exercise group pain intensity, duration and analgesic usage decreased significantly from baseline and this was significantly different to the no treatment group.
Yang 2016	Korea / 40 women	Yoga postures: 10 cycles of surya namaskara for 15 min and then performed shavasana for 5 min of relaxation. Next, five cycles of cat, cobra, and fish yoga poses were performed for 10 min. Finally, the participants performed yoga nidra for 30 min. Yoga nidra was performed in shavasana. This was done for 1 h once a week.	No treatment	12 weeks	Pain intensity (VAS), at end of intervention Pain duration (hours) at end of intervention Moos Menstrual Distress Questionnaire (MMDQ) at the end of the intervention. Adverse events	Menstrual pain intensity and distress decreased significantly in the yoga group compared to the no-treatment group. No participant reported adverse events.

### Study interventions

Four studies examined the effect of heat [46–49], 14 studies examined the effect of self-administered acupressure [50–63] and five studies the effect of low intensity exercise, either stretching or yoga postures [64–68]. Two of the acupressure studies [59, 60] used auricular acupressure, pressure on certain parts of the ear thought to correspond to different organ systems, while the remainder used points located on the body.

#### Location

The majority of the studies were performed in Iran, with five studies [54, 55, 59, 60, 62] performed in Taiwan, two studies [46, 47] performed in the USA, one [48] in Turkey, one [63] in Hong Kong and one [68] in Korea.

#### Control groups

Eighteen studies had two arms, three studies [48, 51, 58] had three arms while two studies [47, 54] had four arms. Control groups were clinically heterogeneous. A pharmaceutical analgesic control, such as ibuprofen, paracetamol or mefenamic acid, was used in nine studies [46–49, 51, 53, 58, 61, 65]. Sham acupressure was used in seven studies [50, 52, 56–60]. No treatment controls were used in six studies [48, 63, 64, 66–68], prescribed rest was used in two studies [54, 62], education on self-care was administered in two studies [51, 55] and placebo pills and unheated patches were used in one study [47].

#### Outcome measures

Pain intensity or severity as measured by the VAS was used as the primary outcome in the majority of trials. Composite measures such as The Andersch and Milsom scale [69] was used in three trials [52, 58, 66], the McGill Pain Questionnaire (SF-MPQ) in three trials [49, 51, 63] and the Moos Menstrual Distress Questionnaire (MMDQ) in five trials [54, 55, 59, 63, 70]. Categorical measures of pain relief using a Likert type scale were used in two trials [46, 47]. Pooled effect sizes used all the above measures of pain intensity and severity but not of duration.

All studies measured pain relief either during or at the end of the intervention itself. Studies using heat based interventions measured pain relief or pain intensity only while the heat was being applied in three of the four studies, either over eight [46, 48] or 12 h [47], with one study measuring the outcome 24 h later [49]. Three acupressure studies measured pain intensity scores at 180 min post intervention [50, 56, 57]. The majority of studies using acupressure and all exercise interventions measured pain intensity that represented the entire menstrual period. No studies assessed short, medium or long-term pain relief after the conclusion of the intervention.

Adverse event reporting was limited in the included studies. Only three studies, two using heat [46, 47] and one using exercise [68] reported adverse events as part of their outcome measures. One study on acupressure [56] reported that adverse events were a reason for drop outs in the control group but no further details were provided.

#### Risk of Bias

**Fig. 2:**
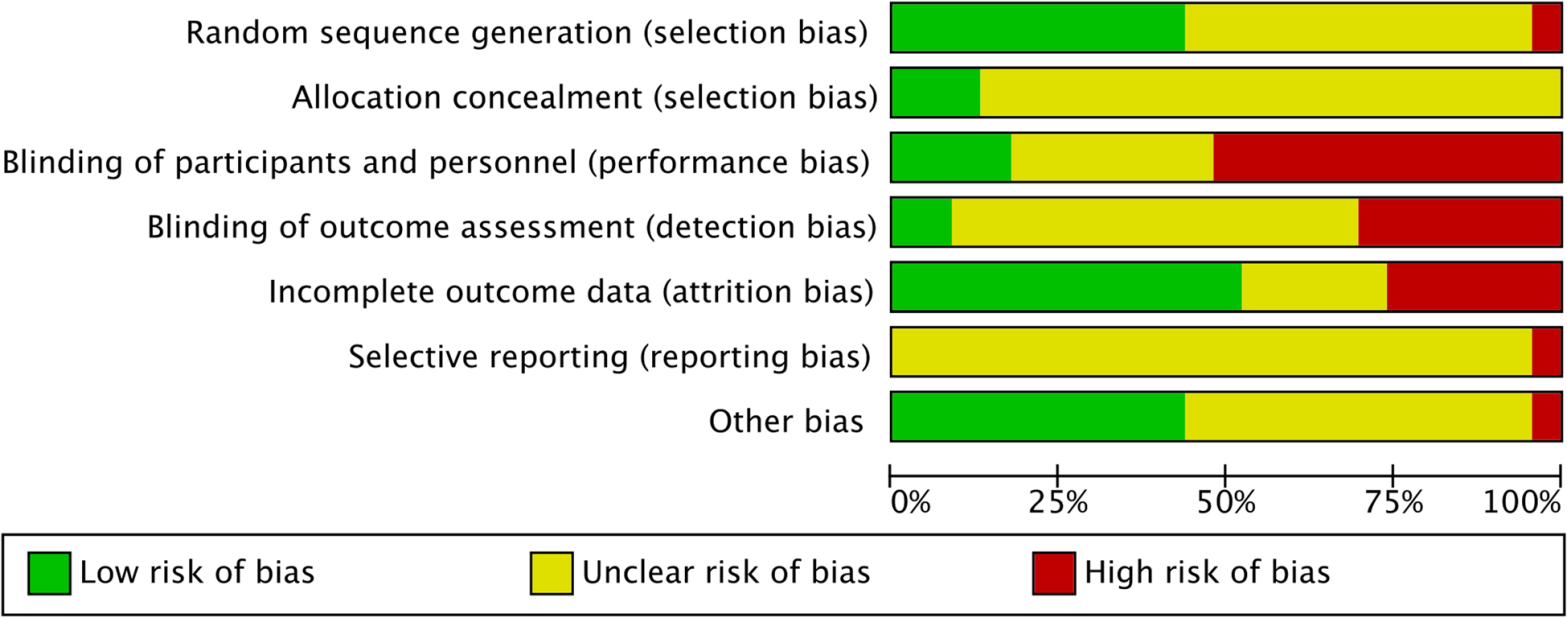
Summary of risk of bias across studies

**Fig. 3:**
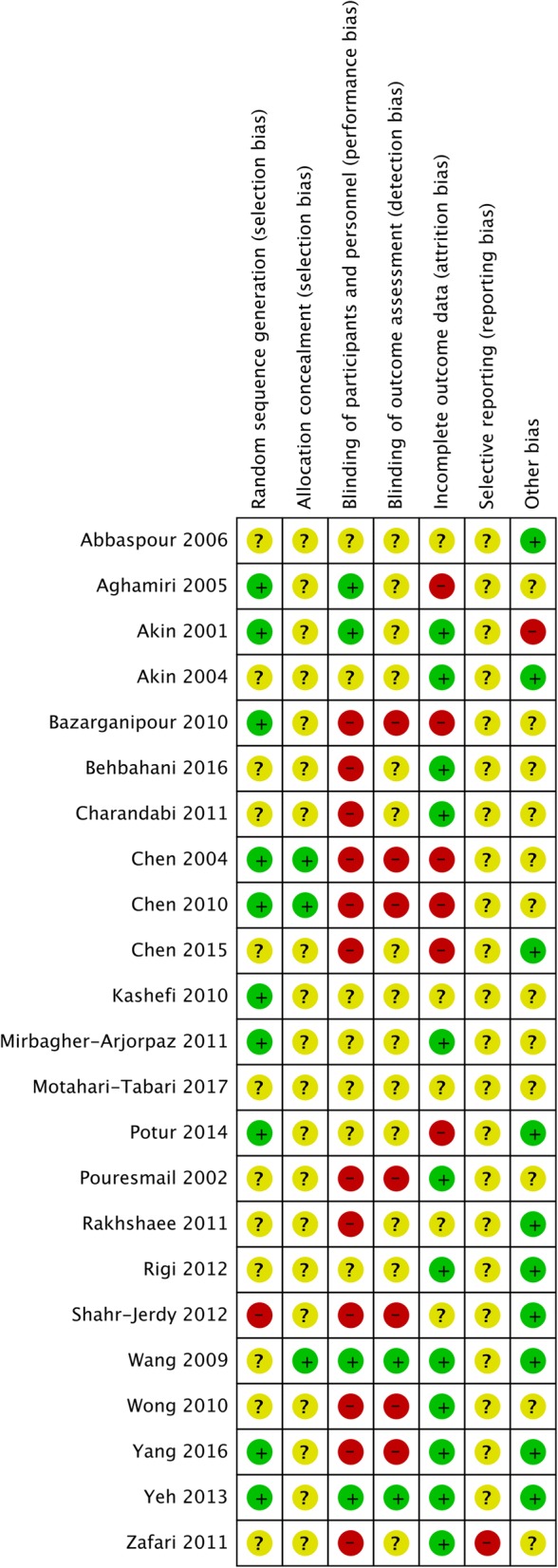
Risk of bias for individual studies

Overall most studies were assessed as having a high risk of bias for at least one domain, and all studies rated unclear for at least two domains, with no studies rating low risk of bias across all domains. Figure 2 shows the overall bias assessment across these domains. Figure 3 shows the risk of bias in each individual included study. Ten studies rated low risk of bias for random sequence generation, 12 studies rated unclear risk and one study was rated high risk of bias. Three studies were at low risk of bias for allocation concealment, with the remaining studies rating unclear. Twelve studies rated high risk of bias for performance bias (blinding of participants and practitioners), with seven studies rating unclear risk of bias and four studies having a low risk of performance bias. Fourteen studies rated unclear for risk of bias relating to detection bias (blinding of outcome measurement), seven studies had a high risk of detection bias, and two studies rated low risk. Twelve studies rated low risk of attrition bias (incomplete outcome data), five rated unclear risk and six were at high risk. Twenty-two studies were at unclear risk of bias for reporting bias, with one study being rated high risk. Ten studies were rated low risk of other bias, twelve had an unclear risk of bias, and one was high risk.

#### Effectiveness of heat therapy

**Fig. 4:**
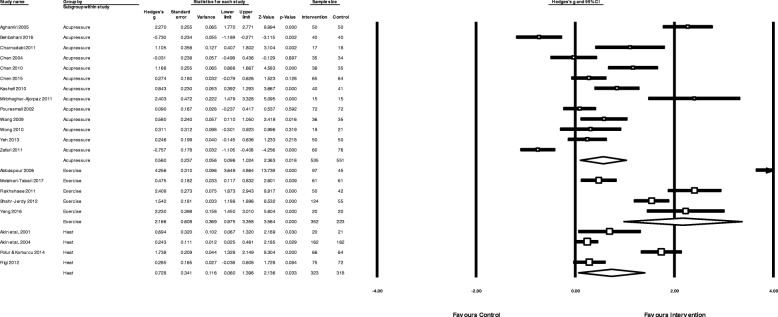
Pooled effects of acupressure, exercise and heat on overall menstrual pain

**Fig. 5:**
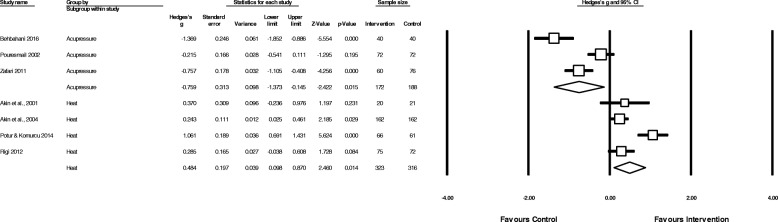
Comparison of the effect of acupressure or heat versus analgesic medication on menstrual pain intensity

The pooled effect of heat therapy (Fig. 4) showed a moderate reduction in menstrual pain (*N* = 4, *n* = 639 g = 0.73, 95% CI 0.06 to 1.40, I^2^ = 92.9,). When compared to analgesic medication control groups only (Fig. 5) there was a small to moderate reduction in pain intensity (*N* = 4, n = 639, g = 0.48, 95% CI 0.10 to 0.87, I^2^ = 76.5).

#### Effectiveness of acupressure

**Fig. 6:**
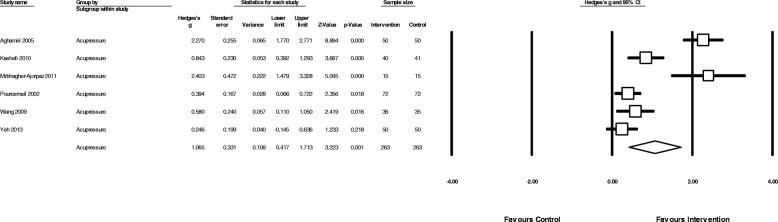
Comparison of acupressure vs sham acupressure on menstrual pain intensity

**Fig. 7:**
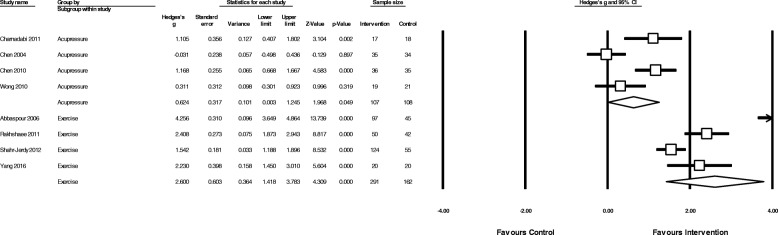
Comparison of acupressure or exercise versus no treatment/usual care on menstrual pain intensity

The overall pooled effect of acupressure (Fig. 4) showed a moderate reduction in overall menstrual pain (*N* = 13, *n* = 1086, g = 0.56, 95% CI 0.10 to 1.03, I^2^ = 92.5,). Acupressure was moderately less effective than analgesic medication (Fig. 5) in reducing pain intensity (*N* = 3, *n* = 360, g = − 0.76, 95% CI -1.37 to − 0.15, I^2^ = 87.2,), but showed a large benefit compared to sham acupressure (Fig. 6) (*N* = 6, *n* = 526, g = 1.1 95% CI 0.42 to 1.17, I^2^ = 91.5) and a moderate benefit vs no treatment (*N* = 4 *n* = 215, g = 0.62 95% CI 0.003 to 1.24, I^2^ = 79.4) (Fig. 7).

#### Effectiveness of exercise

The overall pooled effect of exercise (Fig. 4) showed a large reduction in overall menstrual pain (*N* = 5, *n* = 575, g = 2.2, 95% CI 0.97 to 3.35, I^2^ = 96.7). Exercise was moderately more effective than analgesic medication in reducing pain intensity (*N* = 1, *n* = 122, g = 0.48, 95% CI 0.12 to 0.83) and showed a large reduction in pain when compared to no treatment (N = 4, *n* = 453, g = 2.6, 95% CI 1.41 to 3.78, 4 studies, I^2^ = 94.8) (Fig. 7).

#### Publication bias

There was no evidence of publication bias in trials using heat (*p* = 0.15) or exercise (*p* = 0.23). There was evidence of publication bias in trials using acupressure (*p* = 0.0012). A trim and fill analysis did not result in the removal of any studies and therefore no changes to the effect size. The “fail safe N” for acupressure studies was 140, meaning 140 unpublished studies showing no difference between groups would be required before statistical significance would be > 0.05.

#### Adverse events

Of the three studies reporting on adverse events, only one study on heat reported a single adverse event associated with treatment; a mild reddening of the skin due to heat application [46].

## Discussion

Lifestyle interventions consisting of heat, low intensity exercise and acupressure, appear to provide significant positive reductions in pain intensity, duration and other bothersome symptoms related to menstrual pain.

Low intensity exercise, consisting of yoga and stretching, showed the largest and most consistent positive benefit, with large reductions in pain compared to no treatment and a moderate reduction when compared to ibuprofen, a common over the counter medication used by women for period pain [71]. While the included clinical trials do provide evidence of the effectiveness of yoga and stretching, conflicting results exist from population studies examining the relationship between exercise in general and period pain; with some studies showing no effect of exercise [72], some a positive effect [73] and some a negative effect [74] especially with regards to negative emotional symptoms such as anxiety that can accompany menstruation [75]. Indeed, some women reduce exercise during menses itself [11, 23, 32] while some women prefer to exercise more [11]. This discrepancy between the effectiveness of exercise in the included clinical trials and the community-based research is likely, at least in part, to be due to the definitions of exercise used in these community-based surveys and the weighting given to more aerobic or higher intensity exercise when calculating the amount of exercise performed.

Exercise has often been conceptualised in the public perception as mostly high intensity aerobic activities such as running, swimming or cycling, or resistance training; however lower intensity types of exercise such as yoga, tai chi, Pilates and stretching are gaining greater attention as effective alternatives to the sometimes more injurious traditional forms of high intensity exercise [76]. Interestingly Daley (2008), in a previous review of exercise for menstrual pain, suggested that despite evidence for the effectiveness of exercise broadly for reducing pain, little evidence exists that favours higher intensity (aerobic) exercise over lower intensity options such as yoga and stretching [77]. Different intensities of exercise may operate via different mechanisms. Moderate to high intensity exercise may reduce pain via increasing anti-inflammatory cytokines [78] and by reducing the overall amount of menstrual flow [79], thereby decreasing the overall amount of prostaglandins released. Less intense exercise, such as yoga can reduce cortisol levels [80], which in turn can reduce prostaglandin synthesis [81].

Yoga may have beneficial effects that encompass more than menstrual pain severity. Increased inflammatory markers (such as IL-6 [82] and CRP [83]) are thought to be involved not only in pain but also in mood changes related to menstruation. Mood changes have been shown to be more distressing to many women than the pain itself [33]. There is evidence to suggest yoga may reduce mood changes [84] and this may be mediated via reductions in these inflammatory markers [82].

Heat therapy, using adhesive heat patches on the lower abdomen, showed a moderate improvement in pain intensity compared to placebo patches, placebo pills or no treatment and a small to moderate improvement compared to ibuprofen. Heat may work via both increasing blood flow in the abdominal area [85] and by the ‘gate control’ theory of pain inhibition, where topical heat activates thermoreceptors, inhibiting concurrent nociception and reducing pain signals reaching the brain [46]. While these are promising findings there are several caveats that may reduce heats effectiveness. Most women in the community are unlikely to be able to maintain constant heat at 38 to 40 degrees for 8–12 h using heat packs or hot water bottles, the most common forms of heat used [71]. Heat therapy may also be less effective in those women with greater amounts of adipose tissue on the abdomen, as this acts as a thermal insulator [86]. Therefore, while heat may provide women in the community with reduced pain intensity, it is likely to be of a smaller magnitude than when applied under tightly controlled experimental conditions. Heat, in contrast to exercise, appears to be best reserved for use during menses itself, for rapid, short term pain reduction.

Acupressure showed the most modest benefit in overall pain, with a moderate effect size below heat and unlike heat and exercise, did not show any superiority over analgesic medication. Acupressure is likely to work through a number of pathways, similar to acupuncture, including increasing endogenous opioid release, and increasing uterine blood flow [87]. Our findings on the effect of acupressure are in agreement with other systematic reviews for dysmenorrhea [88] as well as for pain more broadly [89]. While less effective than exercise, acupressure can be easily learnt and simply applied and therefore is a possible adjunct treatment, especially for situations where heat may not be accessible, such as travelling or at school, and for women who do not wish to engage in exercise.

Self-care interventions that women can perform themselves, such as yoga, heat and acupressure are important tools in empowering women to become their own ‘disease manager’ [90]. Women find self-care increases the sense of agency they have over their menstrual cycles, and menstrual pain in particular [33]. Given that low impact physical activity, especially yoga postures, are an accessible lifestyle intervention that confers a number of benefits over the reproductive lifespan including improvements in perinatal depression and menopause [76], it should be recommended, especially in women who receive no or incomplete relief from analgesics. Heat, and to a lesser extent acupressure, may play a role in reducing pain during the menstrual period when performed either prior to, or during menses, and may help in the management of acute menstrual pain.

There are a number of areas which need to be addressed in future research. Given the high prevalence of menstrual cramps, especially in women under 25, researchers have proposed a need to differentiate between normal menstrual cramping from that of dysmenorrhea based on the negative impact on normal activities and inability to manage with analgesics [91]. None of the included trials have made this distinction. This would allow better real-world outcomes, as pain severity increases, so does uptake of self-care practices [92, 93]. Dysmenorrhea commonly results in either absenteeism or reduced classroom performance [2, 9] however trials did not report on these important outcomes. Similarly, given the relationship between increased menstrual flow and dysmenorrhea [94] this factor either needs to be controlled for, or included as an outcome measure in future studies. Additional factors that should be controlled for include; smoking status, BMI, oral contraceptive usage and nulliparity [94]. Finally, given that secondary symptoms such as mood changes and fatigue are very commonly reported [2], and have significant negative impact on women [33] outcome measures should be developed and included that include quantification of these symptoms.

### Strengths and limitations

This systematic review had a number of strengths; we searched for a wide variety of self-care interventions using a broad range of English language databases, had access to translated copies of papers with English language abstracts and undertook the meta-analysis using a ranked order of validated measures. However, there are several important limitations. Firstly, we did not search non-English language databases, therefore if there were publications in journals where there was not an English language abstract these may have been missed from our search, and any papers without an English translation were not able to be included. Secondly, despite grouping together similar interventions there was significant heterogeneity, which was not unexpected given the diversity of interventions and control groups seen in the included studies. This was statistically accounted for in the analysis by using a random effects model, but it does indicate significant differences in the delivery of interventions, and thus we were unable to identify any particular types of intervention (e.g. yoga vs stretching) that were superior. Thirdly, adverse event reporting was poor in most of the included studies. It was unclear if adverse events did not occur or if there was no monitoring of these as part of the study protocol. The included studies on heat suggest minimal risk. Previous systematic reviews have shown that yoga is a relatively safe intervention compared to usual care or exercise [95]. Acupressure, without the use of a device to apply pressure, appears to be a very low risk intervention [96]. Despite the perceived low risk of these interventions, future studies of exercise and acupressure need to include systematic adverse event reporting. Fourth, it was impractical to reduce the risk of performance bias from these interventions. There was an unclear or high risk of bias in the majority of the studies with respect to blinding (either detection bias or performance bias). Due to the self-reported nature of pain, a lack of blinding is likely to induce significant non-specific effects. To overcome these issues, future studies could compare different forms of exercise (e.g. high vs. low intensity, aerobic vs. resistance training, frequency and duration of sessions). This would control for intervention effects while also gaining new insights into types of exercise which may be most beneficial for menstrual pain management. Fifth, the search terms used had a strong focus on mindfulness and relaxation for psychological techniques, therefore papers that used other psychological techniques (such as cognitive behavioural therapy) are likely to have been overlooked.Finally, the risk of bias was unclear for many domains in most of the included studies. This does not necessarily imply low quality, however poor reporting means that caution around the magnitude of these effects would be prudent.

## Conclusion

Given the large proportion of women who get little to no relief from OTC analgesics, our meta-analysis suggests that heat, acupressure or exercise may provide an effective adjunct, or in the case of exercise and heat an effective alternative to, analgesic medication for the management of pain in primary dysmenorrhea. Current research does not address the significant non-specific effects associated with exercise or acupressure interventions. There is a need for future rigorous research designs. Future research on a package of care including some, or all, of these self-care interventions would provide a better understanding of the potential effectiveness and resource requirements of these interventions in a community setting.

## Abbreviations

ACSM: American College of Sports Medicine
COC: Combined oral contraceptive
MMDQ: Moos Menstrual Distress Questionnaire
NRS: Numeric rating scale
NSAID: Non-steroidal anti-inflammatory medication
OTC: Over the counter
PCOS: Polycystic ovarian syndrome
PRISMA: Preferred Reporting Items for Systematic Reviews and Meta-Analyses
SF-MPQ: Short form McGill Pain Questionnaire
VAS: Visual Analogue scale
